# Evaluation of *KRAS*, *NRAS* and *BRAF* hotspot mutations detection for patients with metastatic colorectal cancer using direct DNA pipetting in a fully-automated platform and Next-Generation Sequencing for laboratory workflow optimisation

**DOI:** 10.1371/journal.pone.0219204

**Published:** 2019-07-02

**Authors:** Pauline Gilson, Claire Franczak, Ludovic Dubouis, Marie Husson, Marie Rouyer, Jessica Demange, Marie Perceau, Agnès Leroux, Jean-Louis Merlin, Alexandre Harlé

**Affiliations:** 1 Université de Lorraine, CNRS UMR 7039 CRAN, Institut de Cancérologie de Lorraine, Service de Biopathologie, Vandœuvre-lès-Nancy, France; 2 Institut de Cancérologie de Lorraine, Service de Biopathologie, Vandœuvre-lès-Nancy, France; Sapporo Ika Daigaku, JAPAN

## Abstract

**Background:**

Assessment of *KRAS*, *NRAS* (*RAS*) and *BRAF* mutations is a standard in the management of patients with metastatic colorectal cancer (mCRC). Mutations could be assessed using next-generation sequencing (NGS) or real-time PCR-based assays. Times to results are 1 to 2 weeks for NGS and 1 to 3 days for real-time PCR-based assays. Using NGS can delay first-line treatment commencement and using PCR-based assays is limited by the number of possible analysed targets. The Idylla system is a real-time PCR cartridge-based assay, able to analyse hotspots mutations using one section of FFPE tumour tissue sample. To combine short delays and analysis of a large gene-panel, we propose here a laboratory workflow combining the Idylla system and NGS and compatible with FFPE samples with low tissue quantity. In this study we evaluated and validated the Idylla system for the analysis of *RAS* and *BRAF* mutations by pipetting directly DNA in the cartridge instead of FFPE section as recommended by the manufacturer.

**Materials and methods:**

DNA extracted from 29 FFPE samples from mCRC patients with NGS-characterized *RAS* and *BRAF* mutations were tested with the Idylla KRAS and the Idylla NRAS-BRAF mutation tests to assess sensitivity, specificity, reproducibility and limit of detection of each test.

**Results:**

A 100% concordance was found between NGS and Idylla results for the determination of *KRAS (12/12)*, *NRAS (12/12)* and *BRAF (11/11)* mutations with a sensitivity and a specificity of 100%. The system showed a good reproducibility with CV inferior to 3%. LOD was reached with 2.5 ng of DNA for *KRAS* and *NRAS* mutations and 5 ng of DNA for *BRAF* mutations.

**Conclusions:**

The analysis of *RAS* and *BRAF* mutations using DNA pipetted directly in the cartridge of the Idylla system showed a good sensitivity, specificity, reproducibility and LOD, and can be integrated in a laboratory workflow for samples with few tissue without compromising a further complete tumour characterization using NGS.

## Introduction

Colorectal cancer (CRC) is the third most common cancer in men and the second in women worldwide [1]. Despite novel strategies developed for CRC early detection, 20% of CRC patients are diagnosed at metastatic stage which is associated with 15% five-year survival rate [2–4]. The overall outcome of metastatic CRC (mCRC) has been improved by the addition of anti-epidermal growth factor receptor monoclonal antibodies (anti-EGFR mAbs) to chemotherapy regimens like FOLFOX or FOLFIRI. *KRAS* and *NRAS* tumour mutational status are required for anti-EGFR mAbs prescription since the presence of mutations in exons 2, 3 or 4 of these genes have extensively been described as predictive marker of treatment resistance [5–7]. Mutations in exons 2, 3 and 4 of the *KRAS* and *NRAS* genes occur in approximately 40% and 10% of mCRC respectively. *BRAF* mutations are detected in 8% to 12% of mCRC cases, with a 90% prevalence of *BRAF* p.(Val600Glu) mutations, and are associated with a poor prognosis in this pathology [8,9]. For patients with mCRC, *KRAS*, *NRAS* and *BRAF* tumour genotyping has therefore a major impact on clinical management and genotyping results should be available within 7 working days to ensure a rational first-line treatment selection based on validated molecular data [7]. Genotyping of mCRC is commonly assessed by next-generation sequencing (NGS) which allows the determination of mutational status of a panel of relevant genes. NGS workflow from DNA extraction to results often takes 2 to 4 days depending on the sequencing technology and instrument, but is often closer to 10 days in a clinical testing routine context since all samples are analysed in batch. NGS also requires a reasonable DNA input with an appropriate quality. To shorten the delay from *RAS* testing prescription to the result and also analyse other genes that might be relevant to include the patient in further molecular guided clinical trials, we propose a new strategy that uses both NGS and the fully automated PCR-based Idylla system (Biocartis, Mechelen, Belgium). The Idylla system has been validated for *KRAS* or *NRAS* and *BRAF* hotspot mutations detection in formalin-fixed paraffin-embedded (FFPE) tissue sections [10,11]. To improve the efficiency of our workflow and avoid to deplete the tissue block, especially for small biopsy samples, we have evaluated the ability of the Idylla system to detect *RAS* and *BRAF* mutations by using direct DNA pipetting in the cartridges rather than FFPE tissue sections. In this study, we assessed Idylla sensitivity, specificity, reproducibility and limit of detection (LOD) by using DNA pipetting directly in the cartridge.

## Materials and methods

### Sample selection

A total of 38 extracted DNA from 38 different FFPE tumour samples with a minimal purity of 10% and a NGS-confirmed *KRAS*, *NRAS* or *BRAF* mutation were retrospectively selected among the biological samples collection of Institut de Cancérologie de Lorraine (ICL, Nancy, France). These samples have been collected after routine tumour sequencing for patient cancer management. All patients involved in this study gave their verbal informed consent for the research of *KRAS*, *NRAS* and *BRAF* mutations. The experimental protocol of this study has been approved by the ethical and scientific board of Institut de Cancérologie de Lorraine. All methods were performed in accordance with the relevant guidelines and regulations. All data were anonymized prior to analysis.

Among the 38 patients selected for this study, tumour localisation was colon in 29 (76.3%) patients and rectum in 9 (23.7%). Median age was 71 years (interquartile range 66–80), and M/F sex ratio was 1.7 (Table 1).

**Table 1:** Clinical characteristics of the selected patients.

Patient classification	
Age, year, median (IQR)	71 (66–80)
Sex, female	14 (36.8%)
Primary tumour localisation	
Colon	29 (76.3%)
Rectum	9 (23.7%)
Tumour sample	
Surgery	24 (60%)
Biopsy	14 (40%)
Histology	
Mucinous adenocarcinoma	5 (13.2%)
Lieberkuhnian adenocarcinoma	33 (86.8%)

*IQR* interquartile range

### Analysis of mCRC tumour samples using NGS

DNA was previously extracted from routine mCRC tumour samples. FFPE tissues have been macrodissected after hematoxylin-eosin slide examination and tumour purity determination by a senior pathologist. DNA has been extracted using QIAamp DNA FFPE tissue kit (Qiagen, Hilden, Germany) and DNA concentration measured using the Qubit dsDNA HS assay kit (Qubit 3.0 Fluorometer, ThermoFisher Scientific Inc, Massachusetts, USA). DNA quality was assessed by a cycle of quantification (Cq)-based measurement using the TruSeq FFPE DNA Library Prep QC kit (Illumina, San Diego, USA) following the manufacturer’s recommendations. Delta-Cq (ΔCq) were calculated for each sample using LightCycler 480 Software W UDF 2.0.0 (Roche Diagnostics) by subtracting the Cq value of the sample from the Cq value of the kit’s internal control. ΔCq close or inferior to 0 are retrieved in samples with high DNA quality, whereas ΔCq are greater than 6 for DNA with a quality not suitable for sequencing. Samples with ΔCq included in the [0–6] range have an intermediate DNA quality.

For samples with ΔCq < 6, libraries were prepared using the “Panel INCa” TruSeq Custom Amplicon Library Preparation Kit v1.5 (that includes 16 genes of interest in theragnostic: *AKT1*, *ALK*, *BRAF*, *EGFR*, *ERBB2*, *ERBB4*, *FGFR2*, *FGFR3*, *HRAS*, *KIT*, *KRAS*, *MAP2K1*, *MET*, *NRAS*, *PDGFRA*, *PIK3CA*) (Illumina, San Diego, CA, USA) and then sequenced using MiSeq instrument (Illumina) as previously described [12]. After sequencing, data were analysed using Sophia DDM software v.4.3 (Sophia genetics, Saint-Sulpice, Switzerland). Sequences were aligned using reference genome GRCh37/hg19 and SNV and indels provided by the software with their Variant allele frequency (VAF).

### Analysis of mCRC tumour samples using a fully-automated PCR platform

The Idylla platform is a fully automated, real-time PCR cartridge-based platform which uses microfluidics processing with all reagents on-board and a fluorophore-based detection system. Up to 8 processing units are connected to a console allowing the detection of 8 samples at the same time and independently. Macrodissected FFPE samples sections or extracted DNA are transferred in the single-use cartridge and inserted in the instrument. Several types of cartridges are available (for example, KRAS mutation test, BRAF mutation test, NRAS-BRAF mutation test) and selected according to targeted gene. Inside the cartridge, a combination of heat, high intensity ultrasound, enzymatic and chemical digestion allow cells lysing. Nucleic acids are liberated and ready for subsequent PCR amplification. All steps are automatically performed up to the final report [12].

A Cycle of quantification value (Cq) is calculated for every valid curve by the Idylla console software. Sample Processing Control (SPC) signals reported the amplification of the DNA regions external of the mutational hotspot in each cartridge and testified to sufficient DNA quantity and quality for the analysis. The presence of a mutant genotype is determined by calculating the difference between the SPC Cq and the Cq obtained for the mutant signal. This difference between the control signal and the mutant signal is defined as the ΔCq.

The Idylla *KRAS* mutation test is designed for the qualitative detection of 21 hotspots mutations in exons 2, 3 and 4 of the *KRAS* gene with results available after a 130 minutes run. The Idylla *NRAS*-*BRAF* mutation test is designed for the qualitative detection of 18 mutations in exon 2, 3 and 4 of the *NRAS* gene and 5 mutations in exon 15 of the *BRAF* gene with results available after a 110 minutes run (Table 2).

**Table 2:** *KRAS* mutations detected with the Idylla KRAS mutation test and *NRAS and BRAF* mutations detected with the Idylla NRAS-BRAF mutation test.

Gene	Codon	Protein change	Nucleotide change
KRAS	Codon 12 (exon 2)	G12C	(c.34G>T)
		G12R	(c.34G>C)
		G12S	(c.34G>A)
		G12A	(c.35G>C)
		G12D	(c.35G>A)
		G12V	(c.35G>T)
	Codon 13 (exon 2)	G13D	(c.38G>A)
	Codon 59 (exon 3)	A59E	(c.176C>A)
		A59G	(c.176C>G)
		A59T	(c.175G>A)
	Codon 61 (exon 3)	Q61K	(c.181C>A;c.180_181delinsAA)
		Q61L	(c.182A>T)
		Q61R	(c.182A>G)
		Q61H	(c.183A>C; c.183A>T)
	Codon 117 (exon 4)	K117N	(c.351A>C; c.351A>T)
	Codon 146 (exon 4)	A146P	(c.436G>C)
		A146T	(c.436G>A)
		A146V	(c.437C>T)
NRAS	Codon 12 (exon 2)	G12C	(c.34G>T)
		G12S	(c.34G>A)
		G12D	(c.35G>A)
		G12A	(c.35G>C)
		G12V	(c.35G>T)
	Codon 13 (exon 2)	G13D	(c.38G>A)
		G13V	(c.38G>T)
		G13R	(c.37G>C)
	Codon 59 (exon 3)	A59T	(c.175G>A)
	Codon 61 (exon 3)	Q61K	(c.181C>A)
		Q61L	(c.182A>T)
		Q61R	(c.182A>G)
		Q61H	(c.183A>C; c.183A>T)
	Codon 117 (exon 4)	K117N	(c.351G>C; c.351G>T)
	Codon 146 (exon 4)	A146T	(c.436G>A)
		A146V	(c.437C>T)
BRAF	Codon 600 (exon 15)	V600E	(c.1799T>A; c.1799_1800delinsAA)
		V600D	(c.1799_1800delinsAC)
		V600K	(c.1798_1799delinsAA)
		V600R	(c.1798_1799delinsAG)

In this study, we used extracted DNA directly pipetted in the Idylla KRAS or NRAS-BRAF cartridges, instead of inserting one FFPE section in the cartridge [13].

#### Determination of sensitivity and specificity

Twenty-nine out of the 38 samples included in this study were selected for sensitivity and specificity determination. ΔCq of samples selected range between 0.1 and 5.9, corresponding to high and intermediate DNA quality.

A total input of 10 ng to 25 ng DNA was directly pipetted directly in the cartridge as previously described [13]. Nine samples (#1 to #9) carried an isolated *KRAS* mutation, 9 (#10 to #18) an isolated *NRAS* mutation and 8 (#19 to #26) an isolated *BRAF* mutation. Three samples (#27 to #29) did not carry *KRAS*, *NRAS* or *BRAF* mutations (Table 3). All samples have previously been sequenced using our routine accredited NGS assay. For each sample, tumour purity, variant allele frequencies (VAF) and detailed mutation are described in Table 3.

**Table 3:** Characteristics of samples used to assess Idylla sensitivity and specificity.

Sample	Tumour purity	DNA conc. (ng/μl)	NGS					
			*BRAF*	VAF%	*NRAS*	VAF%	*KRAS*	VAF%
1	20%	13.1	WT		WT		c.351A>T	16.80%
							p.(Lys117Asn)	
2	95%	14.8	WT		WT		c.351A>C	70.96%
							p.(Lys117Asn)	
3	80%	15	WT		WT		c.182A>T	29.39%
							p.(Gln61Arg)	
4	20%	23.4	WT		WT		c.38G>A	13.40%
							p.(Gly13Asp)	
5	65%	27.1	WT		WT		c.34G>A	55.6%
							p.(Gly12Ser)	
6	75%	29.4	WT		WT		c.35G>T	31.80%
							p.(Gly12Val)	
7	70%	53.6	WT		WT		c.34G>A	56.40%
							p.(Gly12Ser)	
8	50%	54.3	WT		WT		c.436G>A	23.80%
							p.(Ala146Thr)	
9	60%	78.8	WT		WT		c.35G>A	57.50%
							p.(Gly12Asp)	
10	50%	53.0	WT		c.34G>A	63.90%	WT	
					p.(Gly12Cys)			
11	70%	57.9	WT		c.37G>C	67.70%	WT	
					p.(Gly13Arg)			
12	70%	72.7	WT		c.181C>A	65.43%	WT	
					p.(Gln61Lys)			
13	30%	87.4	WT		c.182A>T	29.67%	WT	
					p.(Gln61Leu)			
14	40%	88.7	WT		c.35G>A	23.89%	WT	
					p.(Gly12Asp)			
15	50%	49,6	WT		c.181 C>A	37.50%	WT	
					p.Gln61Lys			
16	85%	57,2	WT		c.182 A>G	36.80%	WT	
					p.(Gln61Arg)			
17	60%	41,6	WT		c.182 A>G	29.30%	WT	
					p.(Gln61Arg)			
18	20%	11,5	WT		c.35G>A	39.60%	WT	
					p.(Gly12Asp)			
19	80%	16.9	c.1799T>A	35.50%	WT		WT	
			p.(Val600Glu)					
20	20%	37.8	c.1799T>A	14.70%	WT		WT	
			p.(Val600Glu)					
21	60%	38	c.1799T>A	33.84%	WT		WT	
			p.(Val600Glu)					
22	70%	41	c.1799T>A	30.75%	WT		WT	
			p.(Val600Glu)					
23	30%	55.8	c.1799T>A	10.76%	WT		WT	
			p.(Val600Glu)					
24	70%	61	c.1799T>A	34.61%	WT		WT	
			p.(Val600Glu)					
25	90%	75.2	c.1799T>A	32.70%	WT		WT	
			p.(Val600Glu)					
26	70%	57.2	c.1799T>A	36.80%	WT		WT	
			p.(Val600Glu)					
27	25%	43.4	WT		WT		WT	
28	50%	46.8	WT		WT		WT	
29	60%	58.3	WT		WT		WT	

VAF% = Variant Allele Frequency

NGS was set as the gold standard assay for the calculation of the sensitivity and specificity of each test. Results from samples #1 to 9, #10 to 18 and #19 to 26 were used for the determination of the specificity of *KRAS*, *NRAS* and *BRAF* tests, respectively.

Among these 29 samples, 20 samples with no previously described mutations of *BRAF*, *KRAS* or *NRAS* using NGS were used for the determination of specificity of each assay. Samples #15–18 and #26–29 were used for *KRAS* assay specificity determination, samples #19–29 were used for *NRAS* assay specificity determination and samples #10–18 and #27–29 for *BRAF* assay specificity determination.

All samples were assessed blind-fashioned by a technician using the Idylla system.

#### Determination of the reproducibility

Samples #9, #15, #17 and #24 (described in Table 3) have been used to assess Idylla platform reproducibility. Each sample was run 3 times in the Idylla platform using 25ng of DNA in KRAS mutation test cartridge for samples #9 and #15 and in NRAS-BRAF mutation test cartridge for samples #17 and #24.

For each sample, the reproducibility was estimated using the coefficient of variation (CV%) of Cq obtained for the internal control of each cartridge.

#### Limit of detection

Nine samples out of the 38 samples included in this study have been used to assess the limit of detection (LOD) of the assay. ΔCq of these 9 samples range between 0.4 and 3.3. LOD was defined as the smallest quantity of DNA input that allowed the detection of a mutation in 3 independent samples with different mutations (Table 4). For each test, a total of 7.5, 5.0 and 2.5ng of DNA were pipetted in the cartridge.

**Table 4:** Characteristics of samples used to assess limit of detection.

Sample	Tumour purity	DNA conc. (ng/μl)	NGS					
			*BRAF*	*VAF%*	*NRAS*	*VAF%*	*KRAS*	VAF%
a	60%	41.4					c.35G>T	21,90%
							p.(Gly12Val)	
b	70%	52.4					c.35G>A	37.40%
							p.(Gly12Asp)	
c	70%	71.4					c.38G>A	43.50%
							p.(Gly13Asp)	
d	60%	34.8			c.182A>G	49.18%		
					p.(Gln61Arg)			
e	15%	58.7			c.35G>A	26.13%		
					p.(Gly12Asp)			
f	80%	102.7			c.34G>A	64.62%		
					p.(Gly12Cys)			
g	70%	38.8	c.1799T>A	16.00%				
			p.(Val600Glu)					
h	60%	65.6	c.1799T>A	29.20%				
			p.(Val600Glu)					
i	33%	4.04	c.1799T>A	25.90%				
			p.(Val600Glu)					

Samples #a to c, #d to f and #g to i were used to assess *KRAS*, *NRAS* and *BRAF* tests LOD respectively.

## Results

### Idylla testing and comparison with NGS

Nine *KRAS* mutated samples (#1–9) and eight *KRAS* wild-type samples (#15–18, #26–29) have been tested with the Idylla KRAS mutation test. Nine *NRAS* mutated samples (#10–18), 11 *NRAS* wild-type samples (#19–29), 8 *BRAF* mutated samples (#19–26) and 12 *BRAF* wild-type samples (#10–18 and #27–29) have been tested with the Idylla NRAS-BRAF mutation test. DNA inputs are detailed in Table 5. All tests had a valid result and were 100% concordant with previous NGS results.

**Table 5:** Idylla KRAS and Idylla NRAS-BRAF mutation test performance on extracted DNA.

	DNA input in the cartridge	NRAS-BRAF mutation test		KRAS mutation test
		*BRAF*	*NRAS*	*KRAS*
1	25	/	/	c.351A>C; c.351A>T
				p.(Lys117Asn)
2	25	/	/	c.351A>C; c.351A>T
				p.(Lys117Asn)
3	25	/	/	c.182A>T
				p.(Gln61Arg)
4	25	/	/	c.38G>A
				p.(Gly13Asp)
5	25	/	/	c.34G>A
				p.(Gly12Ser)
6	10	/	/	c.35G>T
				p.(Gly12Val)
7	20	/	/	c.34G>A
				p.(Gly12Ser)
8	25	/	/	c.436G>A
				p.(Ala146Thr)
9	25	/	/	c.35G>A
				p.(Gly12Asp)
10	25	WT	c.34G>A	/
			p.(Gly12Cys)	
11	25	WT	c.37G>C	/
			p.(Gly13Arg)	
12	25	WT	c.181C>A	/
			p.(Gln61Lys)	
13	25	WT	c.182A>T	/
			p.(Gln61Leu)	
14	25	WT	c.35G>A	/
			p.(Gly12Asp)	
15	25	WT	c.181 C>A	WT
			p.(Gln61Lys)	
16	25	WT	c.182 A>G	WT
			p.(Gln61Arg)	
17	25	WT	c.182 A>G	WT
			p.(Gln61Arg)	
18	25	WT	c.35G>A	WT
			p.(Gly12Asp)	
19	25	c.1799T>A	WT	/
		p.(Val600Glu)		
20	25	c.1799T>A	WT	/
		p.(Val600Glu)		
21	25	c.1799T>A	WT	/
		p.(Val600Glu)		
22	25	c.1799T>A	WT	/
		p.(Val600Glu)		
23	25	c.1799T>A	WT	/
		p.(Val600Glu)		
24	25	c.1799T>A	WT	/
		p.(Val600Glu)		
25	10	c.1799T>A	WT	/
		p.(Val600Glu)		
26	25	c.1799T>A	WT	WT
		p.(Val600Glu)		
27	25	WT	WT	WT
28	25	WT	WT	WT
29	25	WT	WT	WT

### Reproducibility

For the four samples tested in triplicate with a DNA input of 25ng a CV% ranging from 1.03 to 2.78% was found (Table 6).

**Table 6:** Reproducibility of Idylla KRAS and NRAS-BRAF assays.

	DNA input (ng)	Type of cartridge	Mutation detected	Cq Run 1	Cq Run 2	Cq Run 3	CV (%)
**#9**	25	*KRAS* mutation test	*KRAS* p.(Gly12Asp) c.35G>A	23.68	24.8	24.91	2.78
**#15**	25	*KRAS* mutation test	*KRAS* WT	27.59	28.71	28.61	2.19
**#17**	25	*NRAS*-*BRAF* mutation test	*NRAS* p.(Gln61Arg) c.182 A>G *BRAF* WT	36.2	36.9	36.8	1.03
**#24**	25	*NRAS*-*BRAF* mutation test	*BRAF* p.(Val600Glu) c.1799T>A *NRAS* WT	36	37.1	36.9	1.60

CV: coefficient of variation

### Limit of detection

All mutations were detected using a 7.5ng DNA input. All *KRAS* and *NRAS* mutations were found using a 2.5ng input. Of the 3 *BRAF* mutated samples, one mutation has not been detected with a 2.5ng input (Table 7). Overall, estimated LOD was 2.5ng of DNA input for *KRAS* and *NRAS* and 5.0ng for *BRAF*.

**Table 7:** Idylla KRAS and Idylla NRAS-BRAF mutation test limit of detection.

	DNA input in the cartridge (ng)	NGS		
		*BRAF*	*NRAS*	*KRAS*
a	7.5	/		c.35G>T
				p.(Gly12Val)
	2.5	/		c.35G>T
				p.(Gly12Val)
b	7.5	/		c.35G>A
				p.(Gly12Asp)
	2.5	/		c.35G>A
				p.(Gly12Asp)
c	7.5	/		c.38G>A
				p.(Gly13Asp)
	2.5	/		c.38G>A
				p.(Gly13Asp)
d	7.5	WT	c.182A>G	
			p.(Gln61Arg)	
	2.5	WT	c.182A>G	
			p.(Gln61Arg)	
e	7.5	WT	c.35G>A	
			p.(Gly12Asp)	
	2.5	WT	c.35G>A	
			p.(Gly12Asp)	
f	7.5	WT	c.34G>A	
			p.(Gly12Cys)	
	2.5	WT	c.34G>A	
			p.(Gly12Cys)	
g	7.5	c.1799T>A	WT	
		p.(Val600Glu)		
	5	c.1799T>A	WT	
		p.(Val600Glu)		
	2.5	**WT**	WT	
h	7.5	c.1799T>A	WT	
		p.(Val600Glu)		
	5	c.1799T>A	WT	
		p.(Val600Glu)		
	2.5	c.1799T>A	WT	
		p.(Val600Glu)		
i	7.5	c.1799T>A	WT	
		p.(Val600Glu)		
	5	c.1799T>A	WT	
		p.(Val600Glu)		
	2.5	c.1799T>A	WT	
		p.(Val600Glu)		

## Discussion

In this study, we evaluated the ability of the Idylla platform for *KRAS*, *NRAS* and *BRAF* mutations detection by directly pipetting extracted DNA in the cartridges instead of introducing FFPE tissue sections as recommended by the manufacturer. With a 100% concordance with NGS results, our data validate that the Idylla system can reliably detect *KRAS*, *NRAS* and *BRAF* mutations by pipetting directly DNA in the cartridge and with a good reproducibility. A DNA input of 2.5 ng and 5 ng were sufficient for mutations detection for *RAS* and *BRAF* respectively.

A threshold of 1% *KRAS* VAF has been reported as clinically relevant and associated with absence of response to anti-EGFR therapy [14]. However, the lowest VAF available in our samples set was 10.7% which did not allow us to determine the minimal VAF that can be detected by the Idylla system using direct DNA pipetting in the cartridge. A similar study which assessed *EGFR* mutations in cytological samples from patients with non-small cell lung cancer extracted DNA showed a limit of detection of 1% [13]. Further investigations with mCRC samples carrying low allele fraction mutation should be relevant to assess the limit of detection of the Idylla system for *KRAS*, *NRAS* and *BRAF* mutations using DNA direct pipetting. Samples included in this study were characterized with NGS which requires DNA with a good or intermediate quality. Therefore, DNA with lower quality were not included is this study. However, Idylla system has previously shown satisfactory sensitivity with poor quality DNA and retrieves samples which failed NGS quality control [12].

Reproducibility of the Idylla system has been previously described on FFPE tissue [15]. Our study validates the reproducibility of the system on extracted DNA.

**Fig 1:**
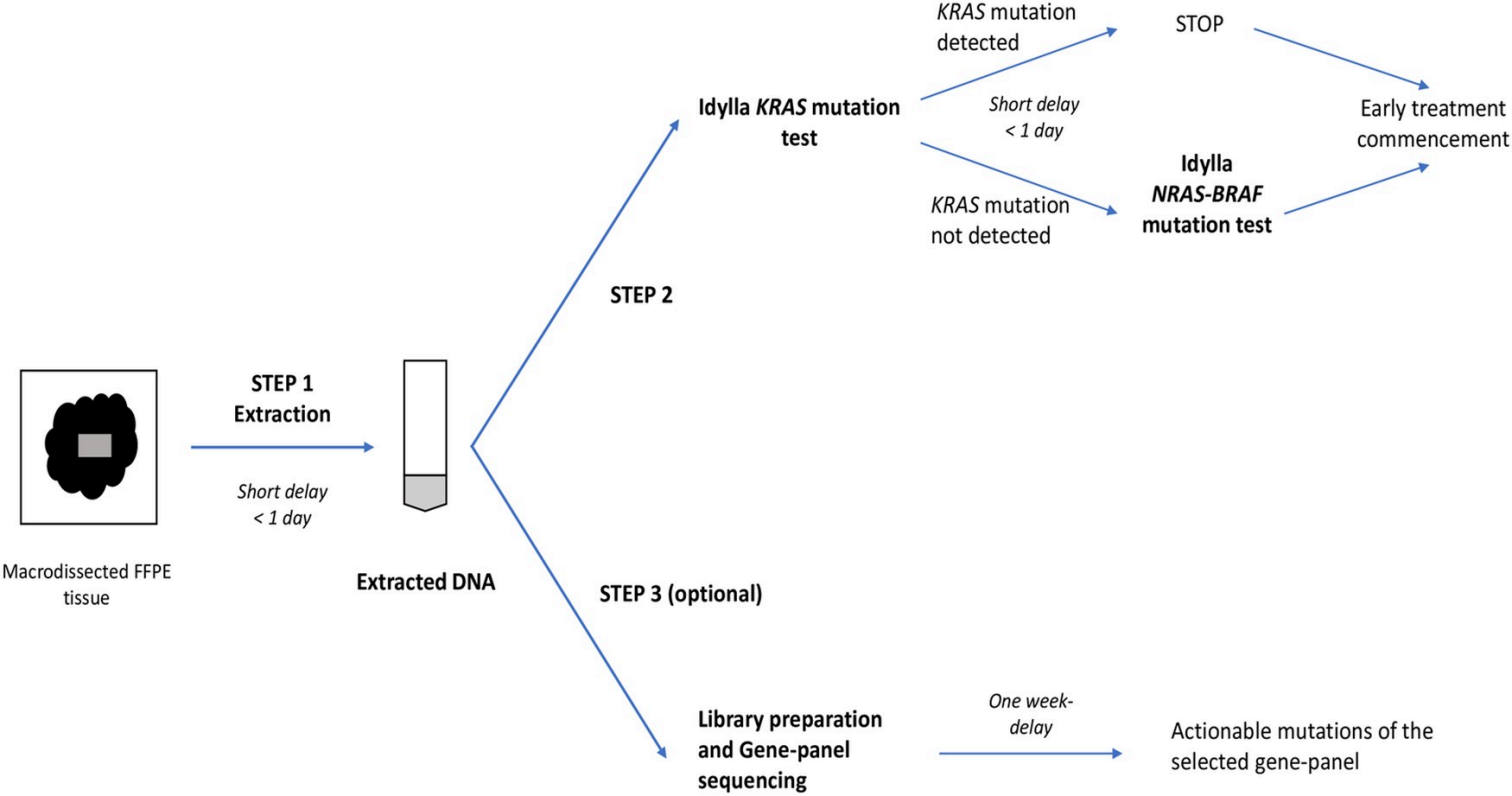
Idylla and NGS combined workflow. First step consists in DNA extraction. Then, extracted DNA is directly introduced in an Idylla KRAS cartridge and if needed in a NRAS-BRAF cartridge (if *KRAS* gene is wild-type). If needed, NGS-libraries can be prepared in parallel.

According to European clinical guidelines, time to results for concomitant *RAS* and *BRAF* genotyping should not exceed 7 working days [7]. With a running-time of less than 2 hours, the Idylla system is compatible with this recommended delay. It was previously shown that the Idylla platform requires lower DNA quality than NGS assays and thus, should be proposed first in a three steps laboratory workflow to have an optimal and early management of patients with mCRC (Fig 1) [12]. First, DNA extraction is performed for all samples. The use of extracted DNA for both methods have the advantage to avoid tumour tissue block depletion, especially for small biopsies with low material. The extracted DNA is secondly directly pipetted in an Idylla KRAS mutation test and the result is available in 2h. If the *KRAS* gene is wild-type, an Idylla NRAS-KRAS mutation test is performed and the result available in 1h30. These two steps can easily be completed in a maximum of 2 days in a routine molecular laboratory, reducing the time to results compared to NGS. The third step if needed consists in the further complete characterization of the tumour and identification of other potent treatment options using NGS. Some NGS platforms need less DNA input than Illumina platform. These alternatives should overcome the question of low material. However, delay to results will always be longer with NGS platform than with Idylla system in first-line.

More than 95% of *KRAS* and *NRAS* mutations are located in codons 12, 13, 59, 61, 117 or 146 and *BRAF* V600E mutation accounts for more than 80% of *BRAF* mutations, therefore the Idylla system that detects only hotspot mutations seems suitable for the molecular analysis of a large majority of patients [16–18]. However, NGS allows the exhaustive DNA analysis of regions of interest with no or few limitations. Whereas only mutations on codons 12, 13, 59, 61, 117 and 146 are validated resistance mutations to anti-EGFR mAbs prescription, non-hotspot *KRAS* or *NRAS* mutations may have also an impact on resistance to anti-EGFR mAbs [16,19]. A broad approach with a panel of genes should be relevant to detect actionable mutations in other genes than *KRAS*, *NRAS* and *BRAF* and discuss cases in multidisciplinary molecular tumour boards [20]. In this context, it should be interesting to have available extracted DNA for further analyses. Indeed, in our routine, most of FFPE samples are sent by external pathology laboratories. Requesting the FFPE block a second time is time consuming and generate additional costs.

In conclusion, using a NGS validated set of samples, we have shown that pipetting DNA directly in the cartridges of the fully automated PCR-based Idylla system gives reliable results for the determination of *KRAS*, *NRAS* and *BRAF* mutations in patient with mCRC. This flexible workflow is suitable when few tumour tissue is available for analysis and drastically shortens delays to results compared to NGS. This workflow allows the further complementary sample analysis using NGS of non-hotspot or potentially actionable mutations in a largest panel of genes.
